# Exploring the utility of bioaerosol metagenomics compared to PCRs for swine pathogen surveillance

**DOI:** 10.3389/frmbi.2024.1439108

**Published:** 2024-10-04

**Authors:** Adrian Muwonge, Priscilla F. Gerber, Bryan A. Wee, Jill Thomson, Jingjing Wang, Patrick G. Halbur, Tanja Opriessnig

**Affiliations:** ^1^ The Digital One Health Laboratory, Roslin Institute and The Royal (Dick) School of Veterinary Studies, University of Edinburgh, Midlothian, United Kingdom; ^2^ Department of Infectious Diseases and Public Health, City University of Hong Kong, Kowloon, Hong Kong SAR, China; ^3^ Scotland’s Rural College (SRUC) Veterinary Services, Veterinary and Analytical Laboratory, Penicuik, Midlothian, United Kingdom; ^4^ Key Laboratory of Animal Epidemiology of the Ministry of Agriculture, College of Veterinary Medicine, State Key Laboratory of Agrobiotechnology, China Agricultural University, Beijing, China; ^5^ Department of Veterinary Diagnostic and Production Animal Medicine, College of Veterinary Medicine, Iowa State University, Ames, IA, United States; ^6^ Vaccines and Diagnostics Department, Moredun Research Institute, Penicuik, United Kingdom

**Keywords:** air, bioaerosol, oral fluid diagnostics, pig farm, surveillance

## Abstract

**Introduction:**

Pathogen introduction and transmission at the farm, regional, or national level are associated with reduced animal welfare and negative impacts on herd economics. Ongoing infectious disease surveillance, active or passive, is therefore of high importance. For optimal resolution, each pig is sampled individually, for example by collecting blood or nasal swabs. In recent years, oral fluids have become very useful for population surveillance at the pen level. Another alternative is sampling the air to capture pathogens circulating across the entire barn via bioaerosols.

**Objective:**

This study aimed to examine the potential utility of bioaerosol metagenomics for pathogen detection on pig farms.

**Methods:**

Bioaerosols via automated air sampler, and oral fluid via pen-based ropes, were collected from each of two Scottish indoor pig farms. All samples were subjected to conventional routine bacterial isolation. Total genomic nucleic acids were extracted for PCR screening for three pig DNA viruses, three bacterial *Mycoplasma* species and an RNA virus. Illumina shotgun metagenomic sequencing was also conducted.

**Results:**

Oral fluids contained more DNA compared to bioaerosol samples. DNA integrity exhibited limited impact on PCR or sequence yield. While *Streptococcus suis* could be cultured from a single oral fluid sample, reads mapped to *S. suis* were detectable in all metagenomic samples. Other bacterial pig pathogens, including *Mycoplasma hyorhinis*, *M. hyopneumoniae* and *M. hyosynoviae*, were detected in oral fluid and aerosols by PCR and metagenomics. One of the two farms was PRRSV positive, and the virus was detectable via PCR in oral fluids but not in bioaerosols. Antimicrobial resistance (AMR) gene profiles had less variation between bioaerosols and oral fluids. Some identified AMR genes had strikingly similar abundance overall.

**Conclusion:**

Overall, these findings indicate that there is potential utility of bioaerosol metagenomics for pathogen surveillance on pig farms; however, more research is needed for technical and cost optimization to allow for routine pathogen detection on livestock farms.

## Introduction

Respiratory diseases are a major challenge to pig health and production efficiency (Opriessnig et al., 2011; Chae, 2016). The majority of the pigs raised today for pork production are housed indoors for reduced mortality, improved average daily gain, more uniform growth rates, and reduced expenses for feed, water and treatments. A disadvantage of being housed in a large population includes the rapid spread of new pathogen strains causing varying severities of morbidity and mortality in the pigs. Respiratory organisms are commonly transmitted via aerosol within and between farms (Hu et al., 2023).

Knowledge of the causes, consequences and susceptibility of pigs to infectious respiratory disease has grown in recent years. The term porcine respiratory disease complex (PRDC) is now used to describe the multifactorial problem (Thacker, 2001). PRDC is almost always polymicrobial in origin (Opriessnig et al., 2011). The relative importance of different infectious agents varies by pig age, farm type and geographic region. Interactions between these factors can be complex, with additive or synergistic effects; some pathogens can cause primary disease, while others may require co-infection to cause disease (Hansen et al., 2010). The multifactorial causes of PRDC represent an obstacle to effective disease control.

Air is composed of two fractions: (1) bioaerosols (short for biological aerosols), which contain fungi, pollen, bacteria and viruses, released from terrestrial and marine ecosystems into the atmosphere (Srikanth et al., 2008), and (2) aerosols, which contain particles with a diameter ≤5 μm, that can remain suspended in the air for significant periods of time and include airborne dust, mists, fumes or smoke (Miaskiewicz-Peska and Lebkowska, 2012). The fraction of airborne particles of biological origin is estimated to amount to up to 37%, with an average number of bacteria and fungi suspended in the air of 1.2 × 10^4^ cells/m^3^ and 7.3 × 10^2^ spores/m^3^, respectively (Miaskiewicz-Peska and Lebkowska, 2012).

Different bioaerosol collection methods in pig houses have been reviewed recently (Raynor et al., 2021). Impingers are specially designed bubble tubes used for the collection of air through nozzles into a liquid collection medium in which particles may be captured as air bubbles through the liquid. In contrast, cyclones use the inertia of particles in air rotating through a passage to capture the particles on the surface of the passage via centrifugal forces. Some samplers combine impingement and cyclonic collection in a single sampler. Impactors utilize inertia to collect particles by turning a particle-filled airstream so that larger particles with higher inertia are captured on a surface. Electrostatic samplers apply charge to particles by generating ions in a sampled airstream and then collect the charged particles in an electrical field. Impingers and cyclone samplers are usually preferred for virus detection by molecular-based or culture-based methods, while all sampler types have been used for the detection of bacteria and fungi (Raynor et al., 2021).

Our study aimed to provide a proof of concept for using bioaerosols from pig farms to detect known pathogens in comparison to oral fluids using standard laboratory methods, i.e. PCR, bacterial isolation, and metagenomics from pooled indexed samples. In effect, the sequencing component of the project can be considered analogous to an environmental metagenomics experiment.

## Materials and methods

### Sampling sites

Two Scottish grow-finish pig farms separated by 200 meters and a solid fence and under different ownership, were selected for the sample collections in this study. An aerial picture of the two farms with the basic building outlines on each farm is provided in **Supplementary Figure S1**. The two farms, Farm A and Farm B, each with two pig barns (A1 and A2 and B1 and B2), were visited for sample collection in August 2014.

Both farms regularly received pigs at approximately 4 weeks of age from different breeding herds. Each batch consisted of approximately 150 pigs and filled a single barn. The average pig age at slaughter was 22-26 weeks. The vaccination history of the pigs was unknown to the staff present on the farms at sample collection, and vaccines were not given after arrival. There were no reports of respiratory or enteric diseases in the farms when the sampling for this study occurred. Farm B also produced chickens, which were housed in a separate barn. Details on the pigs and barns, such as the number of pigs, the age of the pigs, and pens and flooring, are listed in **Supplementary Table S1**. Ventilation and airflow were slightly different for each barn, and details are summarized in **Supplementary Table S2**.

### Sample collection details and overall number of samples obtained and used for further analysis

The sample collection started in farm A with barn A1 followed by barn A2. Next, samples were collected in farm B barn B1, and finally, samples were collected in barn B2. New personal protective equipment (PPE), including hooded coveralls and rubber boots, was used for farms A and B. The sampling team used disposable plastic boots and gloves for each barn. Overall, we collected eight bioaerosol samples including four air1 and four air2 samples (one for each barn and for each air sampler for a total of eight samples) as outlined in **Table 1**. All samples were tested using genomic sequencing, PCR and bacterial isolation.

**Table 1:** Overall numbers of samples obtained in each barn and oral fluid samples used for further analysis.

Farm	Barn	air1	air2	Overall number of oral fluid samples collected	Oral fluid samples analyzed for metagenomic analysis
A	A1	1	1	4	1
	A2	1	1	3	1
B	B1	1	1	5	1
	B2	1	1	3	1
**Total**		**4**	**4**	**15**	**4**

Air1 refers to samples collected using Flir BioCapture 650^®^ air sampler, air2 refers to samples collected with Coriolis^®^ Microbiological air sampler.

### Oral fluid collection

On both farms and in each barn, pens were randomly selected for oral fluid collection. Sampling was performed using an unbleached three-strand cotton rope with a diameter of 16 mm. The rope was affixed at shoulder height of the pigs to feeding troughs for individual pen collection in barns A1, B1 and B2 or on horizontal bars, separating two pens so animals from both pens had access to the rope for barn A2. The collection period lasted approximately 20 min, after which the saturated portion of the rope was placed in a disposable single-use plastic bag. Oral fluid was manually squeezed from the rope within a plastic bag and decanted into a sterile 50 ml centrifuge tube, which was placed on ice in an insulated box for transport to the lab.

### Bioaerosol sampling

**Figure 1:**
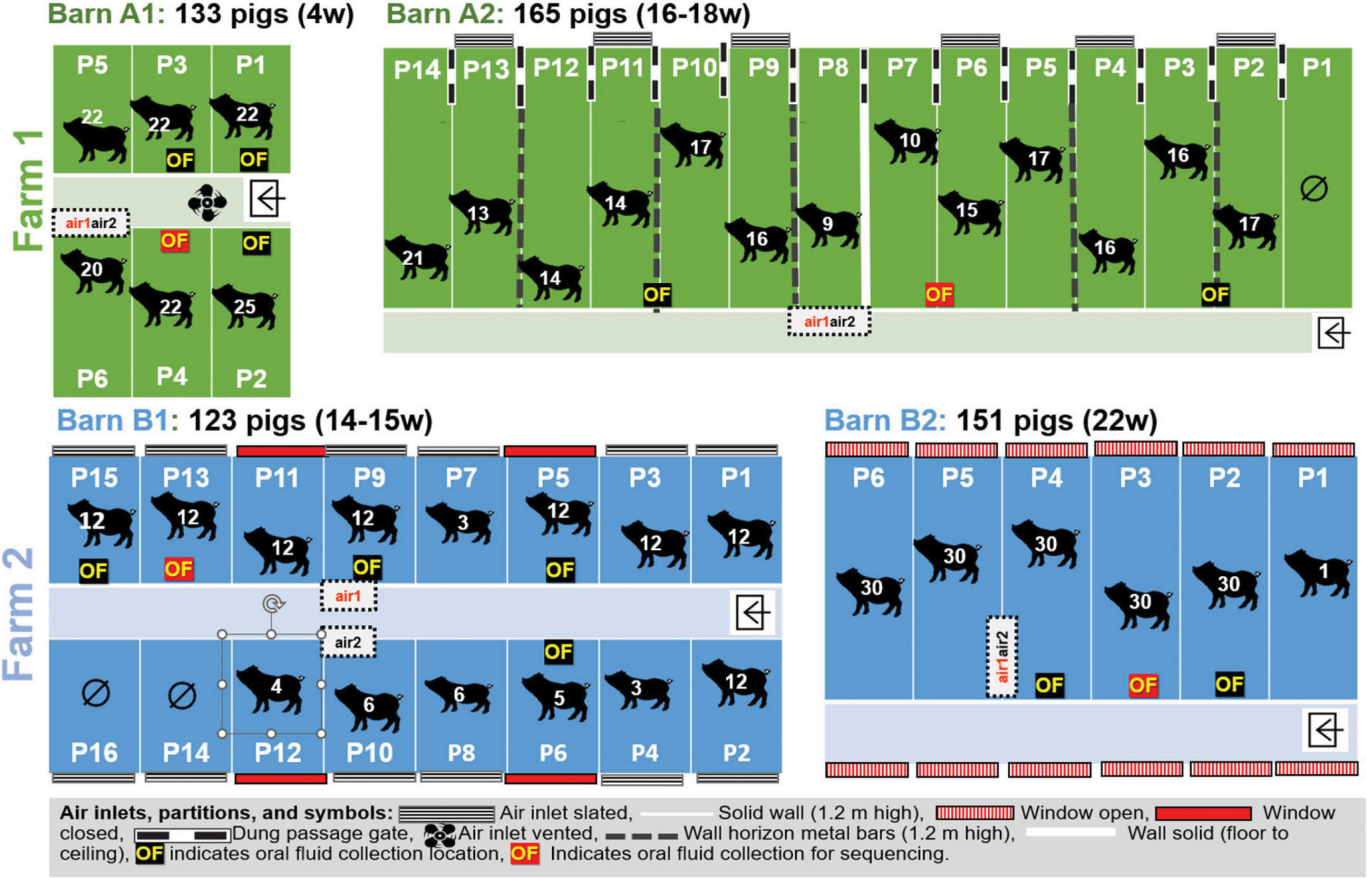
Farm layouts. Farm A (on top in green) and Farm B (on the bottom in blue).

Two different air samplers were used and compared. The Flir BioCapture 650^®^ air sampler (supplied by Southern Scientific Ltd, Henfield, UK), designated as air1 sampler here, is an impactor sampler that collects aerosolized biological particles in the 1-10 µm diameters using a rotating impactor technology and concentrates them into a buffer solution, maximizing the viability of these airborne particles. After collection, a sample is automatically deposited in an easily removable sample vial for subsequent antalysis. The sampler uses a disposable collection cartridge that houses the buffer solution and sample fluid. The Coriolis^®^ Microbiological Bioaerosol sampler (supplied by ACOEM Air Monitors, Glasgow, UK), designated as air2 sampler here, is a cyclonic technology-based machine in which biological particles are collected and concentrated in a buffer. Both Bioaersol samplers were positioned side by side, except in barn B1 where they were placed about 1 m apart and were run simultaneously during each sample collection, as depicted in **Figure 1**. In barn A1 the samplers were placed on top of the wall of pen 6. In barn A2 they were placed at a height of approximately 1.5 m and 1 m from the front of pen 8 (**Figure 1**). In barn B1 the samplers were placed on top of feeders at the height of 1.4 m in pens 10 and 9, respectively, and in barn B2 they were placed on the top of the wall separating pens 4 and 5 at the height of approximately 1.2 m (**Figure 1**). The air1 sampler was run for 15 min with an airflow of 200L/min. The air2 sampler was run for 10 min with an air flow of 300L/min. The eluate from each sampler was stored on ice for 1 h until arrival at the laboratory.

### Sample processing

On arrival in the laboratory, the 15 oral fluids were centrifuged at 1417*g* (2515 rpm) for 20 min, aliquoted, subjected to bacterial isolation and detection of viral and bacterial DNA by PCR assays. The remaining aliquots were stored at -80°C. Four randomly selected oral fluid samples were further processed for genomic sequencing. Specifically, oral fluid samples from farm A1 (pen 4) and A2 (pen 6/7), and also in farm B1 (pen 13) and B2 (pen 3) were used. The eight air samples were aliquoted, and subjected to bacterial isolation and detection of viral and bacterial DNA by PCR assays. The remaining aliquots were stored at -80°C and later further processed for genomic sequencing.

### Bacterial isolation

To detect respiratory bacteria present in the samples, a routine screen for *Lactobacillus pleuropneumoniae*, *Pasteurella multocida*, *Glaesserella parasuis*, *Streptococcus suis*, *Bordetella bronchiseptica, Actinobacillus suis*, and *Salmonella* sp. was performed using sheep blood agar plates (Sigma-Aldrich, Irvine, UK), MacConkey agar (Sigma-Aldrich), and Xylose Lysine Deoxycholate (XLD) agar (Sigma-Aldrich) on refrigerated samples of oral fluid and air samples by a Veterinary Diagnostic Laboratory (SRUC Veterinary and Analytical Services, Penicuik, UK). All cultures were incubated at 37°C for 20 h followed by 10°C for 3 h, followed by assessment and incubation for a further 24 to 48 h in the event of no growth or sparse growth. Other culture plates that were used included Columbia agar plates with 5% sheep blood (Oxoid) which were subjected to aerobic incubation (5-10% CO_2_), plus Oxford Staph streak (NCTC 6571 *Staphylococcus aureus*), Columbia agar with chocolate horse blood (Oxoid) with 5-10% CO_2_ incubation, and MacConkey agar without salt (Oxoid) and Edwards agar (Oxoid) both under aerobic incubation. Salmonella Rappaport (Oxoid) and Selenite (Oxoid) enrichments were used for 20 h under aerobic incubation, followed by culture on Brilliant green and MacConkey agar for another 20 h.

### Detection of viral and bacterial DNA and RNA by PCR assays

DNA was extracted from all eight air samples and the 15 oral fluids collected using 200 µl from each sample further processed using the QIAmp DNA mini kit (Qiagen, Manchester, UK) according to the manufacturer’s instructions, and the DNA was eluted in 100 µl of water. DNA extracts were used to determine the presence of the following viruses using PCR assays previously described. For the detection of porcine circovirus type 2 (PCV2) DNA, a generic real-time PCR was used (Opriessnig et al., 2003). For the detection of porcine parvovirus (PPV) and differentiation of genotypes, a real-time PCR panel composed of a duplex real-time PCR for PPV1 and PPV2 and a triplex real-time PCR for PPV3, PPV4 and PPV5 were used (Opriessnig et al., 2014). Torque teno sus virus (TTSuV) was detected using a duplex real-time PCR to differentiate TTSuV1 and TTSuV2 (Xiao et al., 2012). Two in-house assays based on protocols provided by the Iowa State University Veterinary Diagnostic Laboratory (Ames, Iowa, USA) were used to detect *Mycoplasma hyosynoviae* and *Mycoplasma hyorhinis*. In addition, a commercially available real-time PCR assay (*Mycoplasma hyopneumoniae* Genesig^®^ Standard Kit, Pimerdesign™ Ltd) was used to test the samples for the presence of *Mycoplasma hyopneumoniae.* For all assays, samples with a cycle threshold (ct) equal to or greater than 39 was considered negative. Appropriate positive and negative controls were included in all assays. In addition, samples were also tested for porcine reproductive and respiratory syndrome virus (PRRSV) at SCRUC. A duplex real-time PCR targeting the PRRSV species 1 and 2 (Qiagen Virotype PRRSV RT-PCR) was used for PRRSV detection in RNA extracts. A sample with a ct ≥ 37 was considered negative.

### Genomic sequencing

Eight bioaerosol samples (one air sampler for each farm and barn) and four oral fluid samples were used. We selected oral fluid samples close to the position of the air samplers. A phenol-chloroform extraction of DNA was done. In brief, 0.2 ml of each air sample was transferred in each of five Eppendorf tubes (5 separate tubes were processed for each sample at the same time) with 460 μl STE buffer (10 mmol/L Tris-HCl, 1 mmol/L EDTA, 100 mmol/L NaCl, pH 8.0), 20 μl 0.6% SDS, and 20 μl 200mg/μl pKA (200mg/μl), the contents were mixed by inversion and incubated for 3 h at 65°C. A total of 700 μl of a 25:24:1 phenol-chloroform-isoamyl alcohol mixture (Sigma Aldrich) was added to the tubes, mixed by vortexing and incubated for 5 min at 4°C. Tubes were spun at 12000 rpm for 15 min at 4°C, and the upper aqueous phase from each tube was carefully pipetted to a new tube. An equal volume of chloroform (Sigma Aldrich) was added to the aqueous layer, gently mixed, and contents were spun at 12000 rpm for 15 min at 4°C. The upper aqueous layer was carefully transferred to a new tube, mixed with 30 μl NaCl (5mol/L) and 1 ml of cold ethanol and incubated at -20°C overnight. The following day, the tubes were spun at 12000 rpm for 20 min at 4°C, the supernatant was discarded, and 1 ml 70% ethanol at room temperature was added to each tube. The tubes were spun at 12000 rpm for 5 min, the supernatant was discarded, and the DNA pellet was air-dried. The DNA was suspended in 50 μl EB buffer (10 mM Tris-HCl pH 8.5) and submitted for sequencing. Shotgun metagenomics sequencing of DNA was done by Edinburgh Genomics at the University of Edinburgh. Briefly, library preparation was done using the NEBNext^®^ Ultra™ II FS DNA Library PREP Kit for Illumina (New England Biolabs GmbH, Frankfurt am Main, Germany). Sequencing was done on a HiSeq^®^ 4000 sequencing system (Illumina, Inc., Cambridgeshire, UK).

### Metagenomic analysis

The sequence files are publicly available on the European Nucleotide Archive (ENA) under study accession number PRJEB63145. The reads were processed using the Chan Zuckerberg Infectious Diseases (CZ ID) Illumina metagenomics (mNGS v7.1) pipeline (Parkinson et al., 2012). This pipeline sub samples the reads to two million and assigns reads to taxonomic categories by aligning to the NCBI nucleotide database (indexed 2021-01-22). We chose the CZ ID (https://chanzuckerberg.zendesk.com/hc/en-us/articles/360034790774-How-do-I-cite-CZ-ID-formerly-IDseq) over the Kraken database to maximize the recall rates of infectious pathogens (Li et al., 2024). The subsampling may not affect the detection possibility of high abundance pathogens.

The taxonomic classification results were downloaded and analyzed in RStudio using Phyloseq (v1.44.0) libraries. Shannon diversity indices were calculated using the plot_richness function and Bray-Curtis disssimilarity index were used for the Principal Coordinate Analysis (PCoA) plot, using the plot_ordination function. Plots were generated using ggplot2 (v3.4.2). Antimicriobial resistance genes in farm dust of pig farms have been reported (Luiken et al., 2022). To maximise the detection of antimicrobial resistance genes, we aligned all the metagenomic sequencing reads, before subsampling, with KMA (v1.3.23) with -bc 0.7 against the Resfinder AMR gene database (updated: 26^th^ October 2022). Abundance values of resistance genes were normalized across samples by calculating the number of fragments per kb per million reads (FPKM). As AMR genes are much shorter than whole pathogen genomes, we decided to maximise sensitivity and use the full read set. As AMR genes also have less variation than whole genomes we expected limited impact of false positive AMR gene hits. To compare the abundance of pathogens in air and oral fluid samples, the air-to-oral fluid abundance ratio was used. This allowed us to assess the differential abundance of a given bacteria or AMR gene recovered from the two sample types.If the bacteria and genes were equally abundant in each sample type then the ratio should be 1. If the ratio is greater or less than 1, then the abundance is greater in air and oral fluid, respectively. In the case of zero reads, the bacteria or AMR gene was absent in one of the sample types. The ratios were then used to visualize the relationships using a forest plot and tile plot with ggplot2. We also compared a within-sample abundance ratio of selected pathogens and compared it within each barn. A Pearson correlation coefficient was used to assess the relationship between the abundance of detected pathogens in air and oral fluid samples.

To assess the differences in alpha diversity of air and oral fluid samples we used Kolmogorov-Smirnov test in the Microbiome package (Microbiome R package) in R. The group-wise microbiome structural variance analysis was done using PERMANOVA (https://microbiome.github.io/tutorials/PERMANOVA.html), and the ANOVA-like differential expression tool (ALDEx2) (Fernandes et al., 2014) for high throughput sequencing data with the level of significance reported as >0.05.

### Statistical analysis of PCR results

To test for asymmetry in the number of positive PCR results between binary matched-paired data the McNemar test was used (JMP 17, SAS Institute Inc., Cary NC, USA). A *P* value < 0.05 was considered significant. Specifically, the number of PCR positive samples in air sampler air1 and air2 were compared and the detection rate air sample cumulative results (air was considered positive if at least one of the two air samples in a barn was positive) versus oral fluid cumulative results (oral fluid was considered positive if at least one oral fluid sample of the 3-5 samples collected in a barn was positive) were compared.

## Results

### Microorganism detection

The bacterial isolation for detection of selected viruses and bacteria in air and oral fluid samples is summarized in **Table 2**. All 15 oral fluid and all eight air samples were screened for respiratory bacteria using various agar plates. One of 15 oral fluid samples was positive for *Streptococcus suis* by bacterial isolation, with no significant bacterial growth identified in the remaining 14 oral fluid samples or the four air samples (**Table 2**). Importantly, a wide range of environmental organisms were isolated, indicating that bacterial isolation worked on the samples, but selected swine respiratory pathogens could not be detected in bioaerosols from the farms investigated.

**Table 2:** Detection of selected pig viruses and bacteria based on total nucleic acid amplification and real-time PCR or bacterial isolation on selected sample types (oral fluid or OF, air) collected from two different farms (A, B) and two barns (1,2) on each farm. Numbers indicate ct values. If a ct value was equal or greater than 39 it was considered negative (NEG).

Location	Sample type	Real-time PCR												Bacterial isolation
		DNA virus								DNA bacterium			RNA virus	
		Porcine parvovirus					TTSuV		PCV2	*Mycoplasma hyopneumoniae*	*Mycoplasma hyorhinis*	*Mycoplasma synoviae*	PRRSV-1	
		1	2	3	4	5	1	2						
**A1**	OF1	**36**	NEG	NEG	NEG	NEG	**38**	**39**	NEG	NEG	**35**	NEG	NEG	NSG+
	OF2	NEG	NEG	NEG	NEG	NEG	**38**	**36**	NEG	NEG	**35**	NEG	NEG	NSG++
	OF3	**38**	NEG	NEG	NEG	NEG	NEG	**39**	NEG	NEG	**36**	NEG	NEG	NSG+
	**OF4**	NEG	NEG	NEG	NEG	NEG	NEG	**38**	NEG	NEG	**36**	NEG	NEG	NSG++
	**air1**	NEG	NEG	NEG	NEG	NEG	**39**	**38**	NEG	NEG	**37**	NEG	NEG	NSG++
	**air2**	**38**	NEG	NEG	NEG	NEG	NEG	**39**	NEG	NEG	**35**	NEG	NEG	NSG+++
**A2**	OF2/3	**28**	NEG	NEG	**38**	NEG	**35**	**32**	NEG	NEG	**31**	**31**	NEG	NSG++
	**OF6/7**	**30**	NEG	NEG	**29**	NEG	**33**	**29**	NEG	NEG	**31**	**30**	NEG	NSG++
	OF10/11	**31**	NEG	NEG	NEG	NEG	**35**	**30**	NEG	NEG	**31**	**33**	NEG	NSG++
	**air1**	**34**	NEG	NEG	NEG	NEG	NEG	**37**	NEG	NEG	NEG	NEG	NEG	NSG++
	**air2**	**37**	NEG	NEG	NEG	NEG	NEG	**39**	NEG	NEG	NEG	NEG	NEG	NSG+
**B1**	OF5	**36**	NEG	NEG	NEG	NEG	**37**	**37**	NEG	NEG	**33**	**33**	**33.6**	NSG++
	OF6	NEG	NEG	NEG	NEG	NEG	**38**	**34**	NEG	**39**	**33**	**34**	**36.2**	NSG++
	OF9	**32**	NEG	NEG	NEG	NEG	**33**	**34**	38	**37**	**32**	**33**	NEG	NSG++
	**OF13**	**37**	NEG	NEG	NEG	NEG	**36**	**34**	NEG	**38**	**31**	**34**	**34.3**	NSG++
	OF15	**36**	NEG	NEG	NEG	NEG	**38**	**35**	NEG	**39**	**35**	**33**	**29.3**	NSG+
	**air1**	**37**	NEG	NEG	NEG	NEG	**39**	**37**	NEG	NEG	**37**	**38**	NEG	NSG+
	**air2**	NEG	NEG	NEG	NEG	NEG	NEG	NEG	NEG	NEG	**37**	NEG	NEG	NSG+
**B2**	OF2	**32**	NEG	NEG	**38**	**33**	**32**	**34**	**38**	**39**	**38**	**33**	**36.8**	Strep. suis*
	**OF3**	**30**	NEG	NEG	**37**	NEG	**36**	**33**	**37**	**39**	**35**	**32**	**32.2**	NSG+
	OF4	**24**	NEG	NEG	**37**	NEG	**33**	**32**	**37**	NEG	**38**	**31**	**31.5**	NSG+
	**air1**	**25**	NEG	NEG	**37**	NEG	**35**	**34**	**37**	NEG	NEG	NEG	NEG	NSG++
	**air2**	**30**	NEG	NEG	NEG	NEG	**39**	NEG	NEG	NEG	**38**	NEG	NEG	NSG+

TTSuV=Torque teno suis virus. To better contrast positive results from negative results and to highlight positive results within a virus type, positive ct values were randomly assigned a coloured background and bold black font for ct values. * indicates *Streptococus suis* islation. NEG= the selected pathogen was not found. Air samples are indicated in blue bold font. Oral fluid samples selected for sequencing are indicatd in yellow font with black background. NSG=non-significant growth was identified on essentially all samples including Bacillus species, Stapholoycoccus species, Streptococcus species, coliforms and others+ indicate amount of bacteria present: 0=none, +=few, ++=low, +++=moderate.

The oral fluids and air samples were tested for *M. hyopneumoniae, M. hyorhinis* and *M. hyosynoviae*, by PCR (**Table 2** **).** Mycoplasma species are known to colonize pigs and can cause disease but are difficult to culture *in vitro*. *M. hyopneumoniae* was detected by PCR only in the oral fluid samples in barns B1 and B2 at low levels (6 of 8 oral fluid samples positive, ct values between 37 – 39). *M. hyorhinis* DNA was detected in oral fluids from all four barns at moderate levels (ct values < 33), and in air samples from barns A1, B1, and B2 at lower levels (ct values > 35). *M. hyosynoviae* was detected in all 11 oral fluid samples from barns A2, B1 and B2 at moderate levels (ct values between 30 – 34), while only the air1 sample from barn B1 was positive for this bacterium (ct value of 38).

Various DNA viruses were targeted by PCR and *Ungulate protoparvovirus 1* (or porcine parvovirus type 1, PPV1) DNA was detected in oral fluid and bioerosol samples on all four barns at varying levels (ct values between 24 and 38) (**Table 2**). *Ungulate tetraparvovirus 3* (also known as porcine parvovirus type 2, PPV2) and *Ungulate tetraparvovirus 2* (also known as PPV3) were not detected by PCR in any sample. PPV4 DNA was detected in oral fluid samples of barn A2 and oral fluid and the air1 sample in barn B2. A single oral fluid sample from barn B2 was PPV5 DNA positive, and only farm B samples were positive for PCV2. Among *Anelloviridae*, Torque teno sus viru*s* 1a (TTSuV-1a) from the *Iotaorquevirus* genus and TTSuV k2a DNA from the *Kappatorquevirus* genus were detected in oral fluid and air samples of all barns, except TTSuV1 was not detected in air samples of B A2 (**Table 2**).

### Comparison of microorganism DNA detection rates in oral fluid and air samplers

Obtained data were used to classify a barn as PCR positive or negative for a microorganism in a given sample type; if any of the 3-5 oral fluids collected from a barn tested positive for a microorganism, oral fluid was considered positive for that given microorganism, if any of the two air samples collected from a barn tested positive for a microorganism, air samples were considered positive for that given microorganism. When testing if there was asymmetry in the detection of microorganisms in a sample type, there was a higher detection of microorganisms in oral fluid compared to air samples (McNemar’s test p < 0.01) as some microorganisms were only detected in oral fluid samples as described in the above section. When comparing both air samplers, the air1 sampler had a numerically higher overall positive detection rate for the selected bacteria and viruses tested by PCR, compared to the air2 sampler (eight air1 PCR positive samples with the paired air2 samples negative for the same microorganisms versus two air2 PCR positive samples with the paired air1 samples negative for the same microorganism), without statistical asymmetry in detection between the samplers (McNemar’s test p = 0.11). Low levels of DNA (median ct value 37, range between 25-39) were detected in PCR positive samples for both air samplers.

### Metagenomics of oral fluid and air samples: a comparison between DNA and sequence read output

DNA was extracted from one oral fluid sample and one air sample from each barn, and quantified using a Qubit 4 fluorometer, and the DNA Integrity Number (DIN) was calculated using TapeStation analysis. DNA recovery was 18-80 times greater from oral fluid (median 8.85 ng/μl) compared to air samples (median 0.55 ng/μl), except for air1-B2 (39 ng/μl) that had nearly five times more DNA than the paired oral fluid sample (8.5 ng/μl) (**Supplementary Table S3**). All oral fluid samples had some level of degradation (DIN = 1), with one oral fluid sample from farm B with moderately intact genomic DNA (DIN = 5.9). It is notable that DNA integrity for air samples could not be quantified (DIN < 1). Shotgun sequencing of both sample types generated an average of 47 million paired reads per sample, giving a total of 380 million high-quality paired-end reads based on our quality control (**Supplementary Figure S2**, **Supplementary Table S4**).

**Figure 2:**
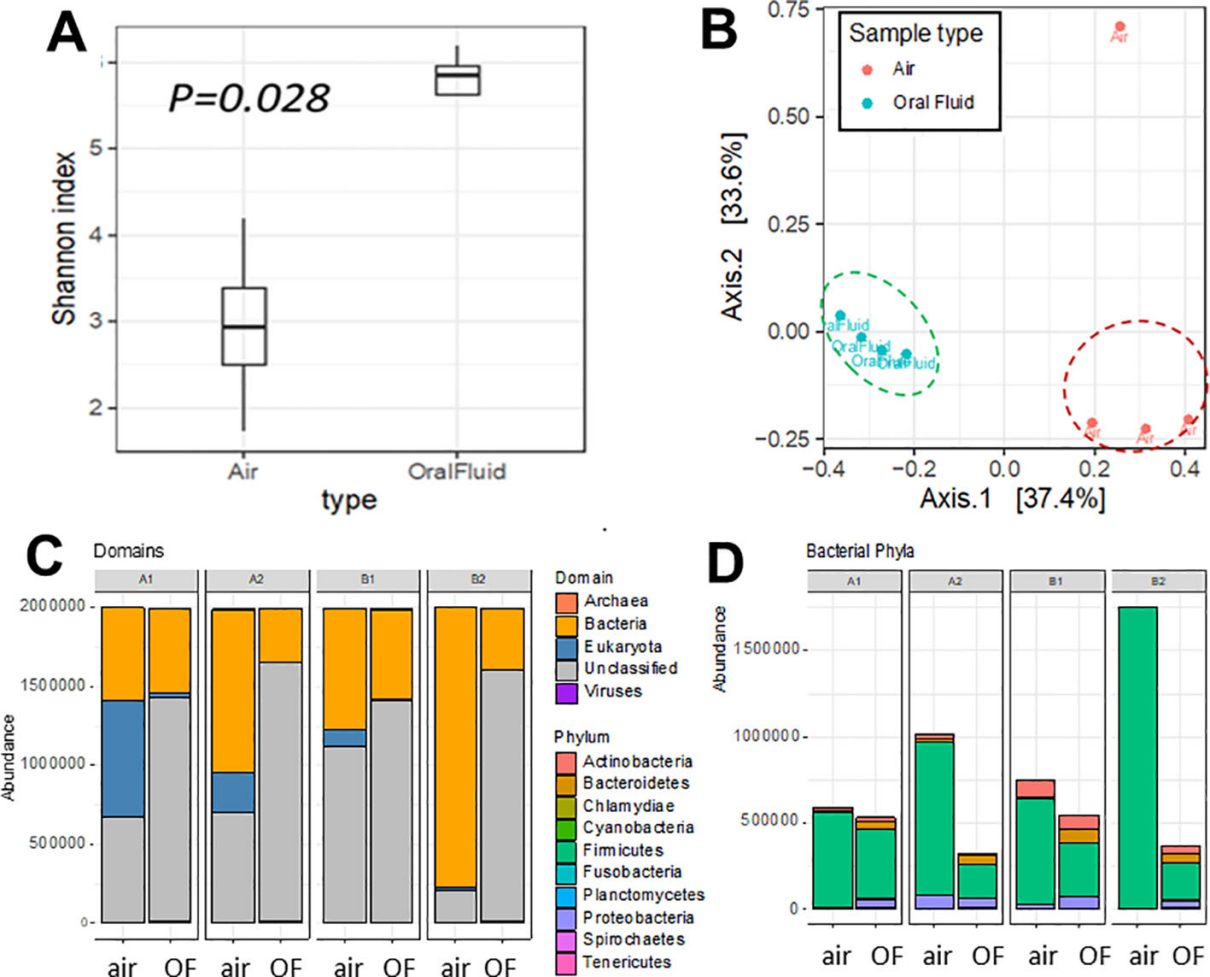
Diversity and taxonomic composition of bioaerosol and oral fluid samples. **(A)** Alpha diversity was calculated using the Shannon diversity index. **(B)** Beta diversity plot shows Axis 1 and 2 of the principal coordinate analysis (PCoA) of the Bray-Curtis dissimilarity index. **(C)** Taxonomic assignment of bioaerosol and oral fluid at the domain. **(D)** Distribution of phyla from the bacterial domain (right) level. Extended data in **Supplementary Table S10** and **Supplementary Figure S4** shows the structural variance between air and oral fluid microbiomes.

An average of 17% of the reads in air and 63% in oral fluid samples mapped to either the pig or human reference genome. These reads were removed before downstream analyses. Using the CZ ID pipeline, which subsamples the reads down to 2 million for computing efficiency and optimized profiling, we generated the taxonomic distribution. The bacteria kingdom dominated the readings in each sample, with the phyla Firmicutes, Proteobacteria, Actinobacteria and Bacteroidetes accounting for 97% of bacteria in each sample (**Figure 2**; **Supplementary Figure S3**). Plant reads were higher in air samples than oral fluid samples (**Supplementary Figure S3**) and mapped to the families *Poaceae* and *Fabaceae*, commonly found in pig feed and the farm environment (e.g., oat, wheat, barley, rice and legumes) (**Supplementary Table S5**). Most viral reads detected belonged to bacteriophages, including uncultured bacteriophages, but also included reads of human pathogens (e.g. human herpesviruses and cytomegalovirus) and porcine viruses considered of low pathogenicity (e.g. porcine mastadenovirus) (**Supplementary Figure S3**, **Supplementary Tables S6**–**S8**).

Oral fluid samples showed consistently higher alpha diversity than the air samples (**Figure 2**; **Supplementary Table S9**). The sample air1 from barn 2 had the lowest level of diversity, likely because of contaminants or particles captured in the air filter based on the much higher DNA concentration values for that sample (**Supplementary Table S9**). Air samples consistently had a higher proportion of bacterial and fungal reads, and the oral fluid samples had a higher proportion of viral reads (**Supplementary Figure S3**). The samples clustered by type (i.e., oral fluid and air) on the PCoA in **Figure 2B**, with sample type explaining 34% of structural variance of the microbiota (PERMANOVA R^2^ = 0.34, p = 0.005) while no significant effect of the barn in which samples were collected was detected (PERMANOVA R^2^ = 0.37, p = 0.24) (**Supplementary Table S10**). There was no evidence of a difference in dispersal between groups (betadisp p = 0.18) (**Supplementary Figure S4**, **Supplementary Table S10**).

### Comparison of PCR detection of microorganisms and metagenomic sequencing

**Figure 3:**
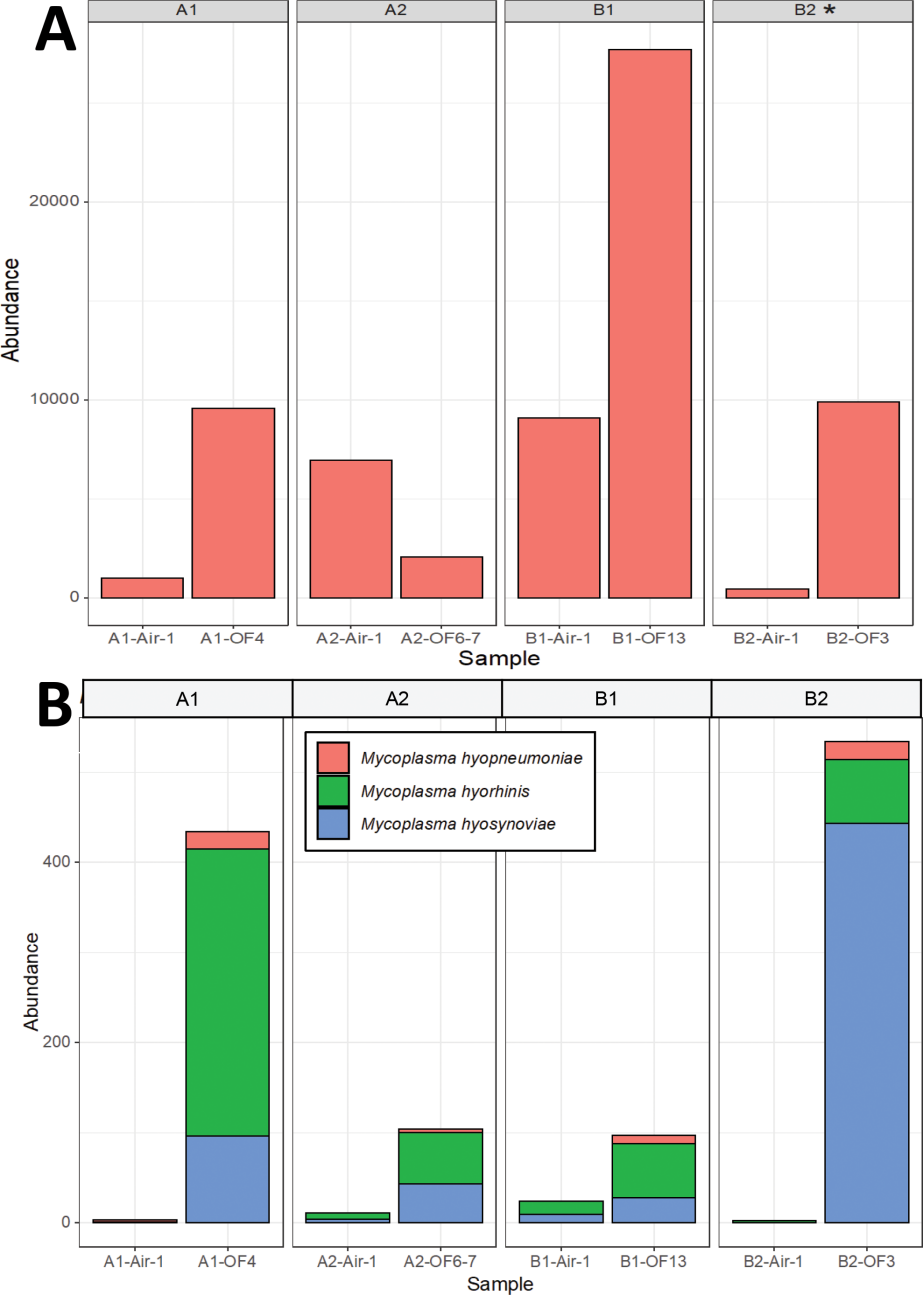
Detection of bacteria using metagenomic sequencing in oral fluids and bioaerosol samples. **(A)** Reads mapped to *Streptococcus* spp. were present across all samples. All samples were also tested for *Streptococcus suis* via bacterial culture (successful isolation is indicated with an *). **(B)** Reads mapped to pig *Mycoplasma* species, including *M. hyopneumoniae*, *M. hyorhinis* and *M. hyosynoviae*. *Mycoplasma* sp isolation was not done as this is not a very sensitive test, and propagation can take up to many weeks and is not always successful (Maes et al., 2021).

There was no consistent association between the detection of specific pathogens using PCR or bacterial culture and metagenomic sequencing. Although only one sample from barn B2 was culture positive for *Streptococcus suis* (**Table 2**), all samples had reads (1,001 to 27,685 reads out of 2M) mapped to *Streptococcus suis* (**Figure 3**), suggesting a low incidence of this commensal microorganism that may cause disease in pigs and humans. *S. suis* was the most abundant member of the genus *Streptococcus* in all samples.

**Figure 4:**
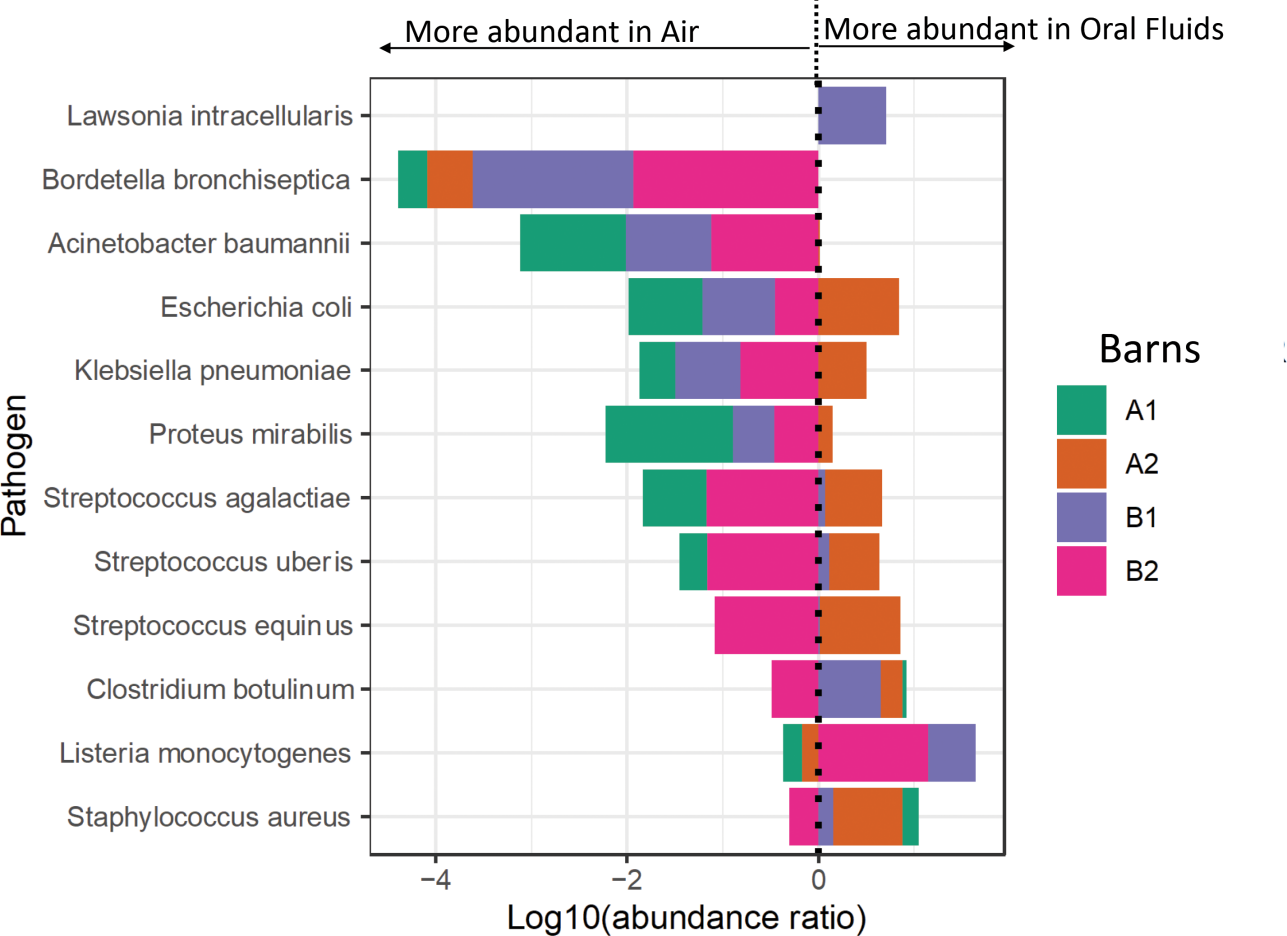
Forest plot of the bacterial pathogens abundance in air and oral fluid samples of the sampled barns. The results to the left of the vertical dotted line (line of no effect) denote the increased chance of microbiota occurrence in air, and the results to the right of the vertical dotted line denote the increased chance of microbiota occurrence in oral fluids. This plot is based on the log10 ratio of abundance of each pathogen in an oral fluid and air samples.

*Mycoplasma hyopneumoniae* was the least abundant of the *Mycoplasma* sp. but still detectable by metagenomics in oral fluid samples from all four barns, although only oral fluids from barn 1 were PCR positive for this pathogen (**Figure 3**). Reads mapped to *M. hyorhinis*, PCR positive in all four barns, were also detected in all four barns by metagenomics, primarily in oral fluid samples (**Figure 3**). *M. hyosynoviae* detected by PCR in oral fluid samples in barns A2, B1 and B2 could also be detected by metagenomics in oral fluid samples, and at low levels in the air samples, with the highest number of reads found in the air sample from barn B1. The commensal low virulence *Mycoplasma flocculare* was also detected by metagenomics in oral fluid samples (A1, B1 and B2). Metagenomic analysis detected potential bacterial pathogens includeding *Acinetobacter baumanii, Bordetella bronchiseptica, Clostridium botulinum, Escherichia coli, Klebsiella pneumoniae, Lawsonia intracellularis, Listeria monocytogenes, Proteus mirabilis, Staphylococcus aureus, Streptococcus agalactiae, Streptococcus equinus* and *Streptococcus uberis* (**Figure 4**; **Supplementary Figure S5**). The abundance ratio for some classes of the pathogens above tended to be higher in oral fluid samples compared to air samples, although some potential pathogens, such as *Bordetella bronchiseptica*, were more readily detected in air samples (**Figure 4**) with a weak positive correlation (R=0.28, P=0.004) between the abundance of the detected pathogens in oral fluids and air samples in the same barn. Nonetheless, overall there were no statistical differences in the abundance of specific taxa at the species, genus or family levels between air and oral fluids (**Supplementary Tables S6**–**S8**). When analyzing for antimicrobial resistance genes, there was a higher detection in air samples compared to oral fluid, particularly those encoding resistance for macrolides, lincosamides, streptogramins (MLS) and tetracyclines (**Supplementary Figures S6**, **S7**).

The only parvovirus detected in the shotgun metagenomic reads was PPV1, with > 5 reads in barn B2. TTSuV and PCV2, detected by PCR, were not detected in the metagenomic analysis.

## Discussion

The use of bioaerosol or air for sampling airborne pig viruses has attracted much interest in recent years (Anderson et al., 2017). A review paper found that air sampling methodologies in pig production have predominately focused on the detection of bacteria and fungi while information on virus burden in the air is limited, with no apparent standardization between different approaches (Anderson et al., 2017).

We compared pathogen detection in the air samples to oral fluid collected via cotton ropes. We also compared the detection of selected pathogens using metagenomics and targeted real-time PCR assays or culture. The motivation for this study was to test the utility of air metagenomics as an alternative method that limits welfare issues associated with surveillance. This study provides proof of concept of the utility of air metagenomics in pathogen detection within pig farming systems. It’s important to note that the methods were not maximized for pathogen detection as no enrichment of samples and metagenomics optimization was done. Critically, more sequence depth is required to detect viral DNA than bacteria in both sample types investigated.

### Comparison between metagenomics, real-time PCR and bacterial culture

Analysis of a selected group of commensal microorganisms with pathogenic potential, such as *Streptococcus suis*, showed that metagenomics could detect reads of such microorganisms where culture was unable to. However, selected viruses were detected by PCR more often than by metagenomic analysis, which is unsurprising given that the samples were not enriched for viral detection. In addition, the abundance of the detected pathogens between methods was not entirely in agreement, which is expected given the normalization process for metagenomics which will affect the relative abundance of specific taxa and differences in minimum detection levels between techniques.

An important consideration when applying molecular methods for pathogen detection using nucleic acid, especially in environmental samples such as air, is that the detected nucleic acids may not represent active infection as it is not possible to ascertain the viability of the pathogen by detection of genomic material. It can, however, serve as an early warning, especially if linked to confirmatory diagnostic investigations within the herd. The DNA detected might be inactivated by on-farm biosecurity practices implemented and in this scenario, DNA detection may be used to detect pathogen circulation.

When considering the comparison of metagenomic abundance between oral fluid and air samples, we used a within and between sample ratio comparison which indicated a weak correlation between the abundance of pathogens detected in oral fluids and air samples in the same barn. Within sample ratio of microorganism abundance might be a better strategy for a reproducible comparison between niches.

### Impact of DNA quality on sequence output

To support the future design and implementation of similar studies, we examined the impact of DNA quality on sequence output. The quality of DNA was evaluated as the amount of shearing, contamination and concentration. Regardless of the low quantity and quality observed, especially for the bioerosol samples, it had a limited impact on the sequence output or the targeted qPCR. To put this in context, we generated an average of a million reads per 0.24 and 1.65ng/µl of air and oral fluid extracted DNA. This tolerance of downstream analyses to DNA variability has been reported elsewhere (Nietsch et al., 2016). The potential to recover high-quality sequence output from relatively low-quality and quantities of DNA was part of the motivation to examine the minimum sequence depth required to robustly detect pathogens. This finding suggests that laboratories with less sophisticated equipment can deploy metagenomics for surveillance without being limited by DNA quality. When using metagenomics, it is important to note that absolute abundances and normalization steps in deep-sequencing may be required.

### Bioaerosol versus oral fluid metagenomics

Our findings indicated a higher detection of viruses in oral fluid than in bioaerosol samples. Among the air samplers, air1 had a numerically higher detection of the tested pathogens by PCR. Each of these sampling techniques inherently contributes to the variability in the microbes detected by metagenomics. However, a small sample size precludes robust comparisons between sample types and air samplers, as only air1 samples were sequenced. Both oral fluid and air sample collection are non-invasive, while oral fluid collection using a rope is more cost-effective, it requires the collection of more samples per barn and a robust cold chain for shipment to the laboratory compared to air samples. Recently, a study compared bacterial diversity and composition of oral fluid, feces and the environment of young pigs (Buiatte et al., 2024). The authors concluded that the oral fluid microbiota of weaned piglets is different (beta diversity) and less diverse (alpha diversity) than the fecal and environmental microbiotas (Buiatte et al., 2024). Air was not investigated in that study. In another microbiome study, tracheal fluids, oral fluids, air, and feces were compared in the late stage of *M. hyopneumoniae* infection in pigs. The study results indicated that air contributed to a greater proportion of bacteria in the trachea compared with feces and oral fluids (Valeris-Chacin et al., 2021).

Porcine viruses may have a quantifiable airborne transmission potential, usually depending on their ability to aerosolize or be carried by dust particles (Verreault et al., 2008), while some bacteria are known to create particles on which other particles are kept airborne (Pandey et al., 2016). From a herd health point of view, microbial load in the air is likely maintained by the number of perspiring/coughing/breathing hosts in a room (Kumar et al., 2021). In a previous study testing the efficacy of air filter systems in preventing pathogen transmission between herds, it became clear that PRRSV and *M. hyopneumoniae* can spread via air (Dee et al., 2010). Conditions for transmission common to both pathogens included cool temperatures, PRRSV or *M. hyopneumoniae* presence in the source population and air and wind direction. PRRSV-positive air days were also characterized by low sunlight levels, winds of low velocity in conjunction with gusts and rising humidity and pressure (Dee et al., 2010).

In the current study, air samples were collected using two liquid cyclonic collectors capable of capturing 200 - 300 L of air per minute, similar to what has been described previously for the RNA viruses PRRSV (Dee et al., 2009) or influenza A virus (IAV) (Corzo et al., 2013). Porcine circoviruses (PCVs) are ubiquitous worldwide, and unlike the groups described above, these are DNA viruses (Gillespie et al., 2009) that can remain infectious in most environments, which makes eliminating them from farms difficult (Patterson et al., 2011; López-Lorenzo et al., 2019). However, the number of reads mapped to PCV2 were low (<5) in 3 oral fluid samples in this study, in concordance with the low detection in the number of samples and DNA levels of this pathogen by PCR.

### Complications of airborne pathogen detection

Most airborne pathogens, particularly viruses, have a short incubation period, and transmission usually occurs days to weeks before the onset of clinical signs, making early detection critical to more effective treatment outcomes. We also investigated the presence of viruses using metagenomics beyond the ones tested by PCR (**Supplementary Tables S6**–**S8**). A wide range of viruses were detected.

Bioaerosol sampling for pathogen detection is an alternative to herd pathogen surveillance; however, it suffers the drawback of low pathogen concentration, which requires specialized instruments, increasing the costs of surveillance (Fronczek and Yoon, 2015). Air pathogens exist as aerosols, dust particles, spores, and a combination of these forms, which means downstream processes are needed to concentrate detectable particulate matter, particularly viruses, diminishing the real-time detection utility of metagenomic studies in air samples; however, some bioaerosol samplers can also be used for PCR detection without extraction and should perhaps be further explored for pig farm sampling. A recently introduced bioaerosol DNA sampler enables high yield and high-quality airborne DNA (Harnpicharnchai et al., 2023), and as an added bonus, the sampler is inexpensive. In addition, there may be a variation in sensitivity to detect particulate forms of a pathogen in the air depending on the collection method and downstream processes used, which may favor bacteria or virus retention. Although strides have been made to improve pathogen detection in the air, very little is available in the literature. Our findings suggest that DNA quality might not impede the use of metagenomics; moreover, we were able to detect sufficient levels to allow direct comparisons with oral fluid samples.

### Limitations of this study

This study is limited by the small number of samples collected from four barns. Shotgun metagenomes were only generated from a single bioaerosol and oral fluid sample per barn. The small number of samples also precludes statistical analyses given the nature of the data, which has a large number of observed taxa. In addition, field blanks for air or oral fluid were not collected. This is important as metagenomics sequencing approaches for low-biomass samples are prone to potential within-lab cross-contamination. Another reason for the inconsistencies is that samples used for sequencing were extracted and processed differently from those used for PCR and culture, which could affect the composition of the bacterial taxa detected. Overall, while valuable insights can still be observed, these constraints must be carefully considered when interpreting the findings of a shotgun metagenomic study with a limited sample size.

## Conclusions

We found that DNA quality had a limited impact on metagenomic sequencing yield and the downstream compositional analysis of the microbiome. A subset of the microbial species and antimicrobial resistance gene patterns observed in air and oral fluids were similar, suggesting that bioaerosol sampling may be useful for detecting selected pathogens. Therefore, these findings support the potential utility of air pathogen detection on pig farms. However, more research is needed for technical and cost optimization to allow for routine utility for pathogen detection on livestock farms.

## Data Availability

The datasets presented in this study can be found in online repositories. The names of the repository/repositories and accession number(s) can be found below: https://www.ebi.ac.uk/ena, PRJEB63145.

## References

[B1] Anderson B. D. Lednicky J. A. Torremorell M. Gray G. C. (2017). The use of bioaerosol sampling for airborne virus surveillance in swine production facilities: A mini review. Front. Vet. Sci. 4, 121. doi: 10.3389/fvets.2017.00121 28798919 PMC5529434

[B2] Buiatte V. Fonseca A. Alonso Madureira P. Nakashima Vaz A. C. Tizioto P. C. Centola Vidal A. M.. (2024). A comparative study of the bacterial diversity and composition of nursery piglets’ oral fluid, feces, and housing environment. Sci. Rep. 14, 4119. doi: 10.1038/s41598-024-54269-5 38374338 PMC10876639

[B3] Chae C. (2016). Porcine respiratory disease complex: Interaction of vaccination and porcine circovirus type 2, porcine reproductive and respiratory syndrome virus, and Mycoplasma hyopneumoniae. Veterinary J. (London Engl. 1997) 212, 1–6. doi: 10.1016/j.tvjl.2015.10.030 27256017

[B4] Corzo C. A. Culhane M. Dee S. Morrison R. B. Torremorell M. (2013). Airborne detection and quantification of swine influenza a virus in air samples collected inside, outside and downwind from swine barns. PLoS One 8, e71444. doi: 10.1371/journal.pone.0071444 23951164 PMC3738518

[B5] Dee S. Otake S. Deen J. (2010). Use of a production region model to assess the efficacy of various air filtration systems for preventing airborne transmission of porcine reproductive and respiratory syndrome virus and Mycoplasma hyopneumoniae: results from a 2-year study. Virus Res. 154, 177–184. doi: 10.1016/j.virusres.2010.07.022 20667494

[B6] Dee S. Otake S. Oliveira S. Deen J. (2009). Evidence of long distance airborne transport of porcine reproductive and respiratory syndrome virus and Mycoplasma hyopneumoniae. Veterinary Res. 40, 39. doi: 10.1051/vetres/2009022 PMC2701181 19379664

[B7] Fernandes A. D. Reid J. N. Macklaim J. M. McMurrough T. A. Edgell D. R. Gloor G. B. (2014). Unifying the analysis of high-throughput sequencing datasets: characterizing RNA-seq, 16S rRNA gene sequencing and selective growth experiments by compositional data analysis. Microbiome 2, 15. doi: 10.1186/2049-2618-2-15 24910773 PMC4030730

[B8] Fronczek C. F. Yoon J. Y. (2015). Biosensors for monitoring airborne pathogens. J. Lab. automation 20, 390–410. doi: 10.1177/2211068215580935 25862683

[B9] Gillespie J. Opriessnig T. Meng X. J. Pelzer K. Buechner-Maxwell V. (2009). Porcine circovirus type 2 and porcine circovirus-associated disease. J. veterinary Internal Med. 23, 1151–1163. doi: 10.1111/j.1939-1676.2009.0389.x PMC7166794 19780932

[B10] Hansen M. S. Pors S. E. Jensen H. E. Bille-Hansen V. Bisgaard M. Flachs E. M.. (2010). An investigation of the pathology and pathogens associated with porcine respiratory disease complex in Denmark. J. Comp. Pathol. 143, 120–131. doi: 10.1016/j.jcpa.2010.01.012 20181357 PMC7094415

[B11] Harnpicharnchai P. Pumkaeo P. Siriarchawatana P. Likhitrattanapisal S. Mayteeworakoon S. Ingsrisawang L.. (2023). AirDNA sampler: An efficient and simple device enabling high-yield, high-quality airborne environment DNA for metagenomic applications. PLoS One 18, e0287567. doi: 10.1371/journal.pone.0287567 37384659 PMC10309600

[B12] Hu Z. Tian X. Lai R. Ji C. Li X. (2023). Airborne transmission of common swine viruses. Porcine Health Manage. 9, 50. doi: 10.1186/s40813-023-00346-6 PMC10619269 37908005

[B13] Kumar P. Kausar M. A. Singh A. B. Singh R. (2021). Biological contaminants in the indoor air environment and their impacts on human health. Air quality atmosphere Health 14, 1723–1736. doi: 10.1007/s11869-021-00978-z 34394766 PMC8346343

[B14] Li Z. Guo Z. Wu W. Tan L. Long Q. Xia H.. (2024). The effects of sequencing strategies on Metagenomic pathogen detection using bronchoalveolar lavage fluid samples. Heliyon 10, e33429. doi: 10.1016/j.heliyon.2024.e33429 39027502 PMC11255660

[B15] López-Lorenzo G. Díaz-Cao J. M. Prieto A. López-Novo C. López C. M. Díaz P.. (2019). Environmental distribution of Porcine Circovirus Type 2 (PCV2) in swine herds with natural infection. Sci. Rep. 9, 14816. doi: 10.1038/s41598-019-51473-6 31616055 PMC6794300

[B16] Luiken R. E. Heederik D. J. Scherpenisse P. Van Gompel L. van Heijnsbergen E. Greve G. D.. (2022). et al: Determinants for antimicrobial resistance genes in farm dust on 333 poultry and pig farms in nine European countries. Environ. Res. 208, 112715. doi: 10.1016/j.envres.2022.112715 35033551

[B17] Maes D. Boyen F. Devriendt B. Kuhnert P. Summerfield A. Haesebrouck F. (2021). Perspectives for improvement of Mycoplasma hyopneumoniae vaccines in pigs. Veterinary Res. 52, 67. doi: 10.1186/s13567-021-00941-x PMC8106180 33964969

[B18] Miaskiewicz-Peska E. Lebkowska M. (2012). Comparison of aerosol and bioaerosol collection on air filters. Aerobiologia (Bologna) 28, 185–193. doi: 10.1007/s10453-011-9223-1 22523449 PMC3321141

[B19] Microbiome R package. Available online at: http://microbiome.github.io/microbiome. (accessed August 5, 2024).

[B20] Nietsch R. Haas J. Lai A. Oehler D. Mester S. Frese K. S.. (2016). The role of quality control in targeted next-generation sequencing library preparation. Genomics Proteomics Bioinf. 14, 200–206. doi: 10.1016/j.gpb.2016.04.007 PMC4996852 27475404

[B21] Opriessnig T. Gimenez-Lirola L. G. Halbur P. G. (2011). Polymicrobial respiratory disease in pigs. Anim. Health Res. Rev. 12, 133–148. doi: 10.1017/S1466252311000120 22152290

[B22] Opriessnig T. Xiao C. T. Gerber P. F. Halbur P. G. (2014). Identification of recently described porcine parvoviruses in archived North American samples from 1996 and association with porcine circovirus associated disease. Vet. Microbiol. 173, 9–16. doi: 10.1016/j.vetmic.2014.06.024 25081955

[B23] Opriessnig T. Yu S. Gallup J. M. Evans R. B. Fenaux M. Pallares F.. (2003). et al: Effect of vaccination with selective bacterins on conventional pigs infected with type 2 porcine circovirus. Vet. Pathol. 40, 521–529. doi: 10.1354/vp.40-5-521 12949409

[B24] Pandey R. Usui K. Livingstone R. A. Fischer S. A. Pfaendtner J. Backus E. H.. (2016). et al: Ice-nucleating bacteria control the order and dynamics of interfacial water. Sci. Adv. 2, e1501630. doi: 10.1126/sciadv.1501630 27152346 PMC4846457

[B25] Parkinson N. J. Maslau S. Ferneyhough B. Zhang G. Gregory L. Buck D.. (2012). Preparation of high-quality next-generation sequencing libraries from picogram quantities of target DNA. Genome Res. 22, 125–133. doi: 10.1101/gr.124016.111 22090378 PMC3246199

[B26] Patterson A. Madson D. M. Schalk S. D. Halbur P. G. (2011). Opriessnig, T.: Establishment and maintenance of a porcine circovirus type 2 (PCV2)-free breeding herd on a site that experienced a natural outbreak of PCV2-associated reproductive disease. J. Swine Health Prod 19, 165–174. doi: 10.54846/jshap

[B27] Raynor P. C. Adesina A. Aboubakr H. A. Yang M. Torremorell M. Goyal S. M. (2021). Comparison of samplers collecting airborne influenza viruses: 1. Primarily impingers cyclones. PLoS One 16, e0244977. doi: 10.1371/journal.pone.0244977 33507951 PMC7842955

[B28] Srikanth P. Sudharsanam S. Steinberg R. (2008). Bio-aerosols in indoor environment: composition, health effects and analysis. Indian J. Med. Microbiol. 26, 302–312. doi: 10.1016/S0255-0857(21)01805-3 18974481

[B29] Thacker E. L. (2001). Immunology of the porcine respiratory disease complex. Veterinary Clinics North America Food Anim. Pract. 17, 551–565. doi: 10.1016/s0749-0720(15)30006-2 PMC7134923 11692508

[B30] Valeris-Chacin R. Sponheim A. Fano E. Isaacson R. Singer R. S. Nerem J.. (2021). Relationships among fecal, air, oral, and tracheal microbial communities in pigs in a respiratory infection disease model. Microorganisms 9, 252. doi: 10.3390/microorganisms9020252 33513772 PMC7912642

[B31] Verreault D. Moineau S. Duchaine C. (2008). Methods for sampling of airborne viruses. Microbiol. Mol. Biol. Rev. MMBR 72, 413–444. doi: 10.1128/MMBR.00002-08 18772283 PMC2546863

[B32] Xiao C. T. Giménez-Lirola L. Huang Y. W. Meng X. J. Halbur P. G. Opriessnig T. (2012). The prevalence of Torque teno sus virus (TTSuV) is common and increases with the age of growing pigs in the United States. J. Virol. Methods 183, 40–44. doi: 10.1016/j.jviromet.2012.03.026 22484614

