# Supplementary material for: Exploring the utility of bioaerosol metagenomics compared to PCRs for swine pathogen surveillance
Source: Front Microbiomes. 2024 Oct 4;3:1439108. doi: 10.3389/frmbi.2024.1439108 (PMC12993622; doi:10.3389/frmbi.2024.1439108)
Supplement: Supplementary file 4 [file DataSheet4.docx]

# Supplementary material: Exploring the utility of bioaerosol metagenomics compared to PCRs for swine pathogen surveillance

**Farm information including farm layout**

**and barn and ventilation details**


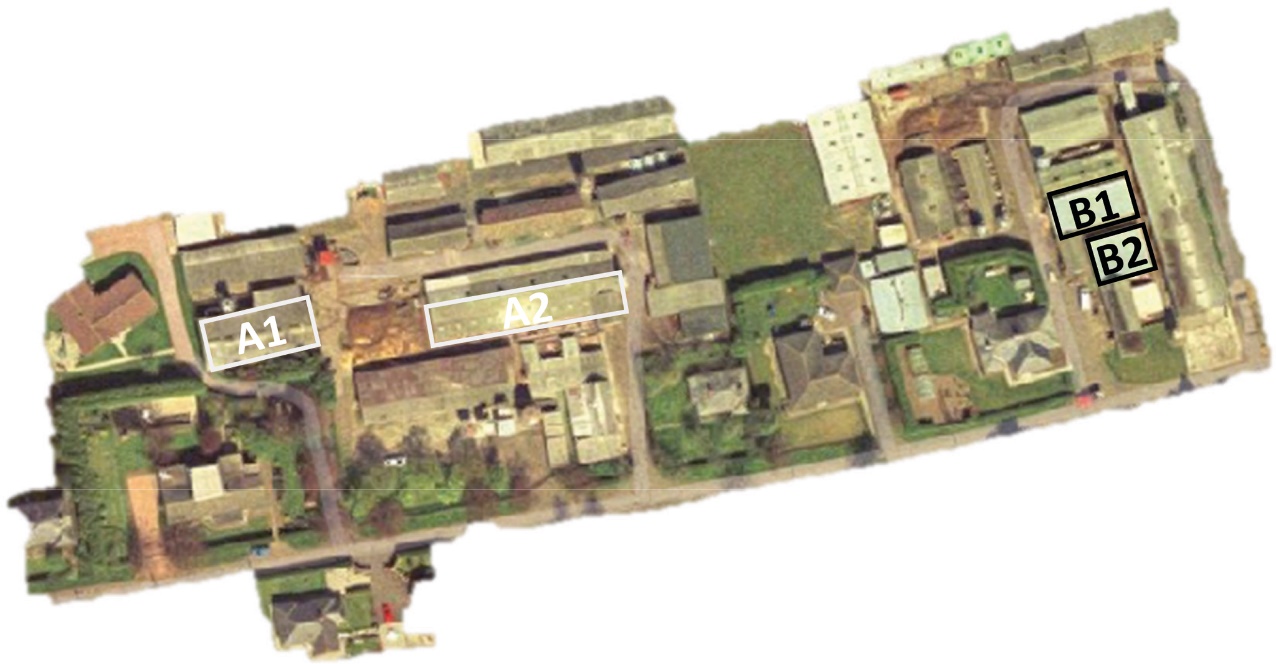


**Fig S1.** **Aerial map of farms A and B.** The investigated barns (1 and 2) are indicated by white boxes (farm A) or by black boxes (farm B). A1 and A2 are two barns on farm A and B1 and B2 are two barns on farm B.

**Table S1.** **Characteristics of the barns investigated.**

| **Barn** | **Pig** | | **Pens** | | | **Partition** | | **Flooring** |
| --- | --- | --- | --- | --- | --- | --- | --- | --- |
|  | ***No.*** | ***Age*** | ***No.*** | ***Size*** | ***m^2^*** | ***Type*** | ***Height*** |  |
| **A1** | 133 | 4w | 6 | 1.6×2.8m | 4.5 | Solid | 1.2 m | Slatted |
| **A2** | 165 | 16-18w | 14* | 1.7×8.0m | 13.6 | Solid  Open (metal bars)  Central wall (concrete) | 1.2 m  1.2 m  Full | Solid with straw |
| **B1** | 123 | 14-15w | 16 | 2.2×4.0 | 8.8 | Solid | 1.2 m | Slatted |
| **B2** | 151 | 22w | 6 | 2.4×9.5 | 22.8 | Solid | 1.2 m | Slatted |

*Each pen has a separate dung area as part of a dung passage which has gates to keep pigs in their pen from the dung passage site during cleaning. w indicates age in weeks.

**Table S2. Ventilation details of the two farms (A, B) and 4 barns (A1, A2, B1, B2).**

| **Farm** | **Barn** | **Air inlet** | **Air outlet** | **Type of ventilation** |
| --- | --- | --- | --- | --- |
| **A** | **A1** | One fan above the door | 6 fans in the ceiling | Natural (stack effect) |
|  | **A2** | Six fans in the long wall | 4 fans in the ceiling | Natural (stack effect) |
| **B** | **B1** | Six slatted fans and two windows on either side of the long wall | 2 fans in the ceiling | Mechanical |
|  | **B2** | Six slatted windows on either side of the long wall | Eaves in the ceiling | Natural (stack effect) |

**Metagenomics of oral fluid and air samples:**

**A comparison between DNA and sequence read output**

**Table S3.** DNA concentration given as ng/µl and integrity number (DIN) for oral fluid and air samples.

| **Barn** | **Oral fluid DNA**  **concentration** | **DIN** | **Air DNA**  **concentration** | **DIN** |
| --- | --- | --- | --- | --- |
| A1 | 7.5 | 1 | 0.4 | <1 |
| A2 | 9.2 | 1 | 0.2 | <1 |
| B1 | 56.1 | 1 | 0.7 | <1 |
| B2 | 8.5 | 5.9 | 39.4 | <1 |

**Sequence read quality and read statistics**


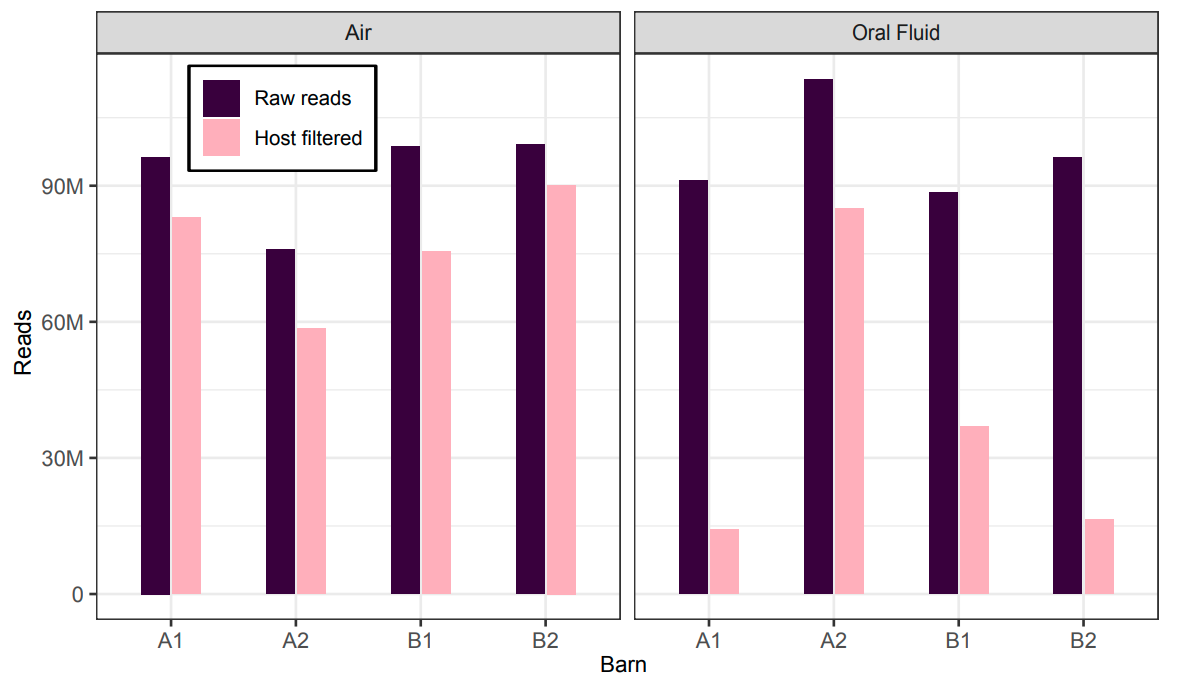


**Fig S2.** Unpaired read counts for metagenomic shotgun sequencing

before and after host (pig and human) sequence filtering.

**Table S4.** Sequencing characteristics and the metagenomic bioinformatic pipeline quality control.

| **Barn** | **Sample ID** | **Sample**  **type** | **Total Reads** | **Passed Phred filter** | **% pass**  **QC** | **Compression**  **ratio** |
| --- | --- | --- | --- | --- | --- | --- |
| A1 | Air-1 | Bioaerosol | 96329580 | 83029833 | 97.5 | 1.15 |
| A1 | OF4 | Oral fluid | 91156148 | 14127499 | 96.3 | 1.13 |
| A2 | OF6/7 | Oral fluid | 113486080 | 85053764 | 97.2 | 1.15 |
| A2 | Air-1 | Bioaerosol | 75994338 | 58534393 | 97.1 | 1.13 |
| B2 | OF3 | Oral fluid | 96234072 | 16347320 | 95.9 | 1.14 |
| B1 | Air-1 | Bioaerosol | 98642222 | 75519057 | 95.5 | 1.16 |
| B1 | OF13 | Oral fluid | 88589696 | 36888250 | 97.6 | 1.14 |
| B2 | Air-1 | Bioaerosol | 99037708 | 90162095 | 95.9 | 1.16 |

**Comparing air and oral fluid**

**detection taxonomic composition**


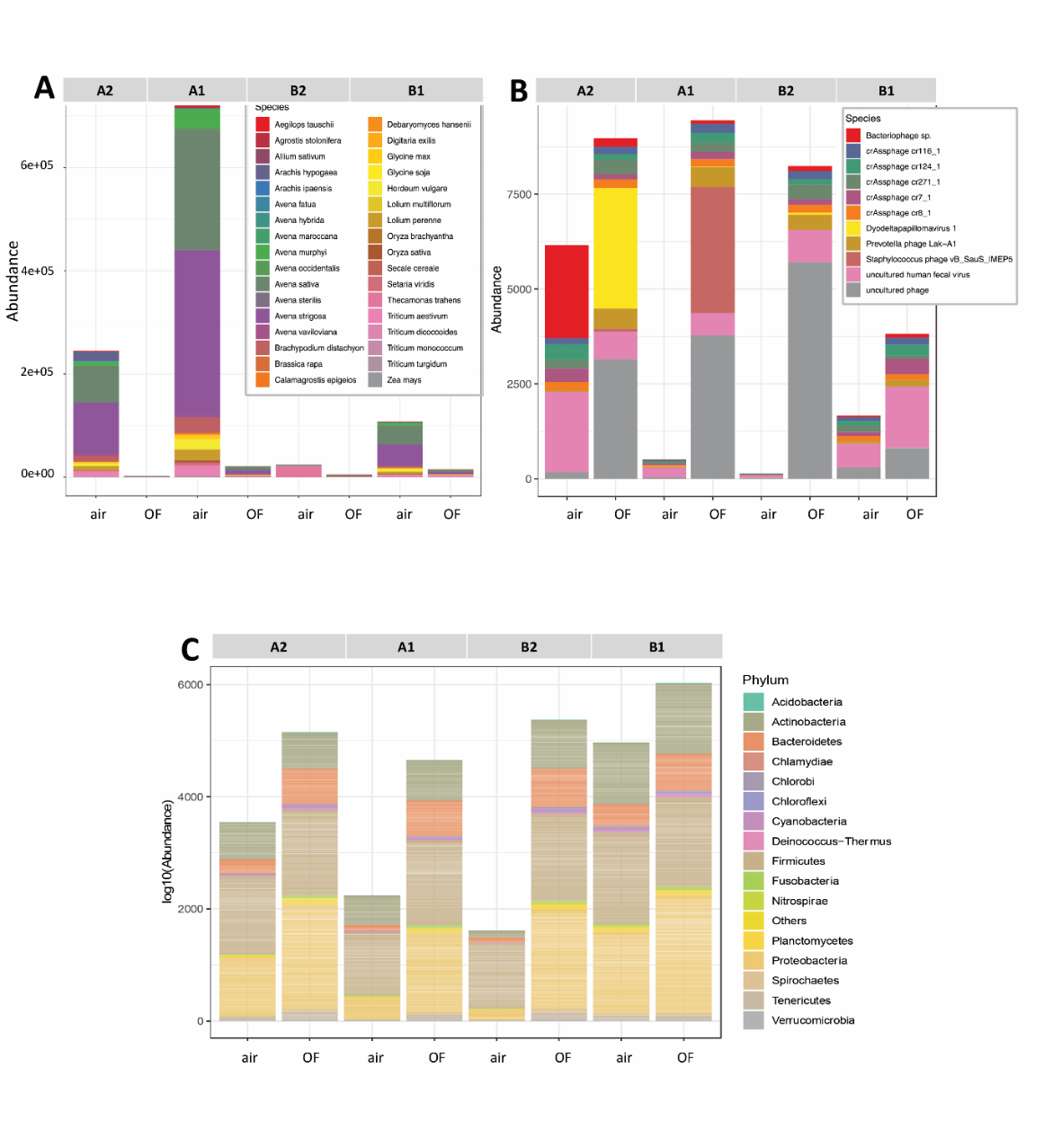


**Fig S3.** Comparing taxonomic composition of air and oral fluid microbiome. **A.** Plant species, Air and oral fluid reads that mapped to eukaryotic organisms, are indicated. **B.** Bacteriophages, reads are shown that correspond to viral DNA. **C.** Bacteria phyla (with log10 abundance) in air and oral fluid samples.

**Detecting animal feed ingredients in air samples**

Air metagenomics has the capability to characterize animal feed, as illustrated in **Fig S3** and allowed us to identify the specific components present in airborne dust particles suspected to originate from pig feed.

We also identified several grass species, including Creeping Bent Grass (*Agrostis stolonifera*), Stiff Brome (*Brachypodium distachyon*), and ryegrass (*Lolium multiflorum* and *Lolium perenne*), all of which are native to Scotland and likely introduced into the barn via airflow. In contrast, Goat Grass (*Aegilops tauschii*), which is not native to this region, is likely a filler material in the feed.

**Table S5.** Eukaryotic DNA detected that map to known pig feed ingredients.

| **Ingredients** | **Taxonomic name** | **Detected in air** | **Pig Feed ingredient** | **Source** |
| --- | --- | --- | --- | --- |
| Maize | *Zea mays* | Yes | Yes | <https://www.thepigsite.com/articles/how-to-farm-pigs-feeding> |
| Rice | *-Oryza sativa*  *-Oryza brachyantha* | Yes | Yes | <https://www.thepigsite.com/articles/how-to-farm-pigs-feeding> |
| Oats | *-Avena murphyi*  *-Avena fátua*  *-Avena Sativa* | Yes | Yes | <https://www.thepigsite.com/articles/how-to-farm-pigs-feeding> |
| Wheat | *-Triticum aestivum* | Yes | Yes | <https://www.thepigsite.com/articles/how-to-farm-pigs-feeding> |
| Soya bean | *-Glycine max*  *-Glycine soja* | Yes | Yes | <https://www.thepigsite.com/articles/how-to-farm-pigs-feeding> |
| Millet | Digitaria exilis | Yes | Yes | <https://www.feedstrategy.com/animal-feed-formulations-library/growing-finishing-pig-feed-formulations> |
| Barley | *Hordeum vulgare* | Yes | Yes | <https://www.thepigsite.com/articles/how-to-farm-pigs-feeding> |

**Differential abundance of taxa in air and oral fluids**

Aldex2 Kruskal-Wallis tests with Bonferroni correction were at the species, genus and family level, as can be seen in the Excel files **Table S6** (Species level), **Table S7** (Genus level) and **Table S8** (Family level). The Aldex2 Kruskal-Wallis tests on the CZ ID calls were done at the species, genus and family level. Nothing is significant after correction for multiple testing; however, some samples are below 0.05 before correction. It doesn’t say anything about the direction of the effect (higher in air or in oral fluid). There were no significant differences between sample types at any level.

**The columns are as follows:**

kw.ep a vector containing the expected p-value of the Kruskal-Wallis test for each feature

kw.eBH a vector containing the corresponding expected value of the Benjamini-Hochberg corrected p-value for each feature

glm.ep a vector containing the expected p-value of the glm ANOVA for each feature

glm.eBH a vector containing the corresponding expected value of the Benjamini-Hochberg corrected p-value for each feature.

**Comparing microbiome diversity indices of**

**bioaerosol and oral fluids**

**Table S9**. Diversity indices of each sequenced sample.

| **Sample ID** | **Inverse Simpson** | **Shannon** | **Fischer** | **Dominance** |
| --- | --- | --- | --- | --- |
| A2-Air-1 | 3.89 | 3.11 | 664.98 | 0.56 |
| A1-OF4 | 30.58 | 5.04 | 915.76 | 0.18 |
| B1-OF13 | 73.44 | 5.82 | 1053.85 | 0.11 |
| B2-OF3 | 55.71 | 5.88 | 1188.78 | 0.14 |
| A1-Air-1 | 3.50 | 2.76 | 491.49 | 0.61 |
| B1-Air-1 | 7.46 | 4.19 | 919.84 | 0.39 |

The table extends **Fig. 4** in the manuscript. It shows that the oral fluid microbiome is much more diverse by all measures. However, the proportion of potentially pathogenic bacteria is higher in the air sample as seen in Fig 6 in the manuscript. This suggests that if optimised for a specific bacterium, the air sample is likely to be more specific.

**Table S10**. PERMANOVA analysis examining the microbiome structural variance.

| **Variable** | **Degrees of freedom** | **R2** | **P-value** |
| --- | --- | --- | --- |
| Barn | 3 | 0.37 | 0.246 |
| Sample type | 1 | 0.34 | 0.0052 |
| Residuals | 3 | 0.27 | - |
| **Total** | **7** | **1.0** | **-** |

The table extends **Fig. 3** in the manuscript. It shows the PERMANOVA output which examines the extent to which the sample type (oral fluid and air sample) and the barn of the pigs explain the structural variance of the microbiome

**Fig S4.** A box-and-whisker plot showing the distance to the sample type centroid in each group i.e. sample type (air or oral fluid).

This plot is an extension of **Table S10** and of shows the “distances” of each sample to the centroid of the group type. The distances are calculated from the PCoA based on the "dissimilarity" used in **Table S10** with betadisper function in the vegan package in. The comparison suggests that air and oral fluid samples do not differ in homogeneity dispersion (Sum of square = 0.04, p=0.18).

**Comparing air and oral fluid**

**detection of bacterial pathogens**


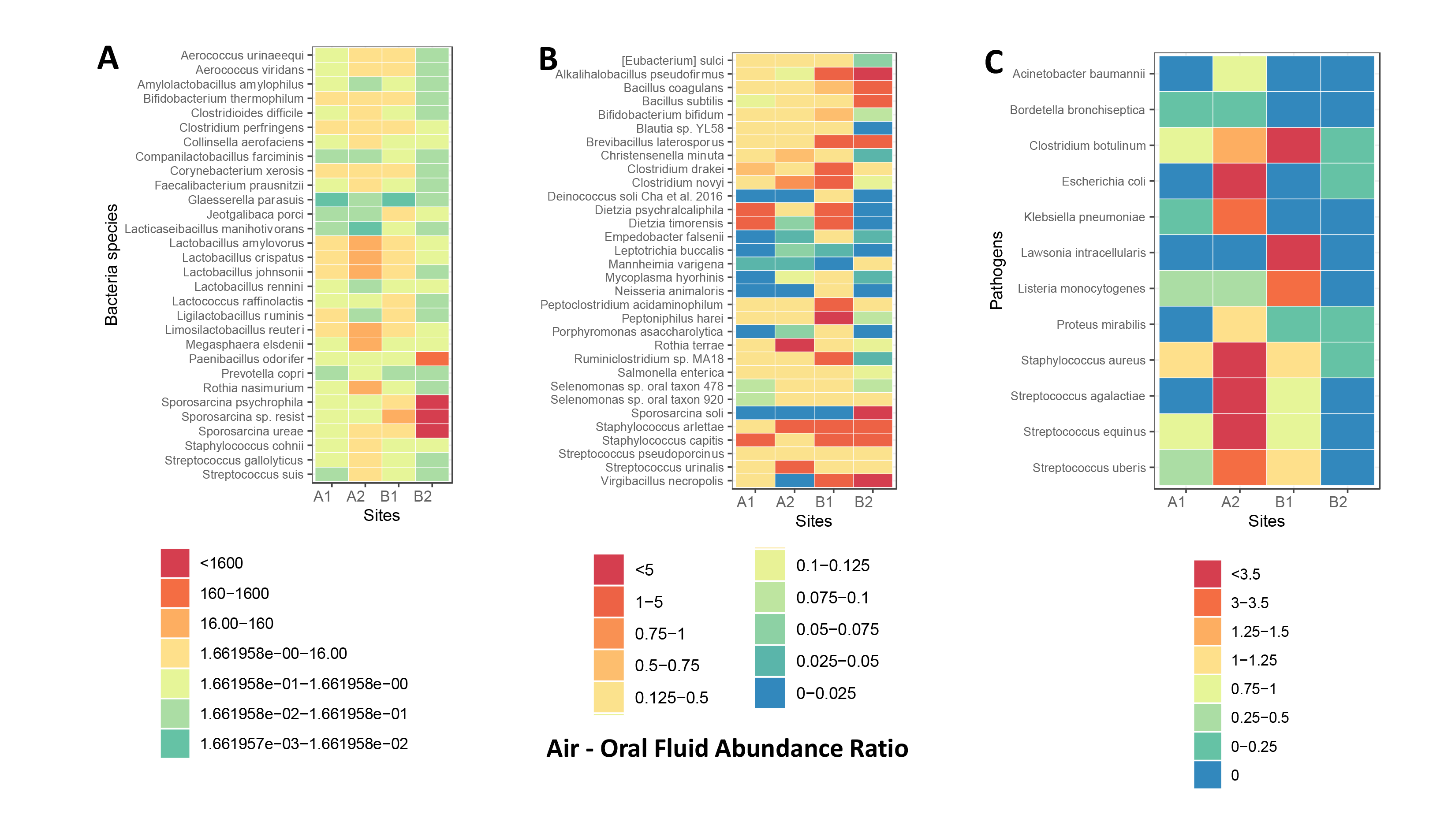


**Fig S5. Air to oral fluid ratios of key bacterial pathogens.** Warmer colours (orange to red) indicate a higher abundance of the-air-to oral fluid ratio (1>) which means the abundance of the given bacteria are greater in air compared to oral fluid, while cooler colours (blue to green) (1<) indicate a low abundance ratio, meaning the abundance of a given bacteria is higher in oral fluid samples compared to air samples. When the ratio is 0, the bacteria is present in only one sample type, while when the ratio is 1, the bacteria is equal-abundant in both sample types. **4A** compares the dominant and **4B** compares the rare bacteria in the microbiome respectively. Finally, **4C** shows 12 common pathogenic bacteria.

**The resistome in bioaerosol and oral fluid samples**

The metagenomic reads were also analysed for antimicrobial resistance (AMR) genes. All pre-subsampled and unfiltered reads were used for this analysis to maximize the sensitivity and the ability to detect reads from shorter and relatively less abundant AMR genes. Alpha-diversity of the AMR genes did not show separation by sample type, unlike the taxonomic data. However, 357 unique AMR genes were identified representing 21 types of resistance phenotypes. On average, AMR genes were detected in higher relative abundance in air samples, particularly those encoding resistance for macrolides, lincosamides, streptogramins (MLS) and tetracyclines **(Fig S6, Fig S7)**. For the most abundant genes, air samples captured the same AMR genomic context as the oral fluid samples **(Fig S6)**. This was true for macrolide resistance genes such as mef(A) and aminoglycoside resistance gene ant(6)-Ia. However, the most abundant AMR gene, *tet*(O/W) was more abundant in air vs oral fluid samples.

**Fig S6.** A comparison between air and oral fluid reads per kilobase pair mapping to 14 antibiotic classes. The combined class included genes that encode resistance across classes.


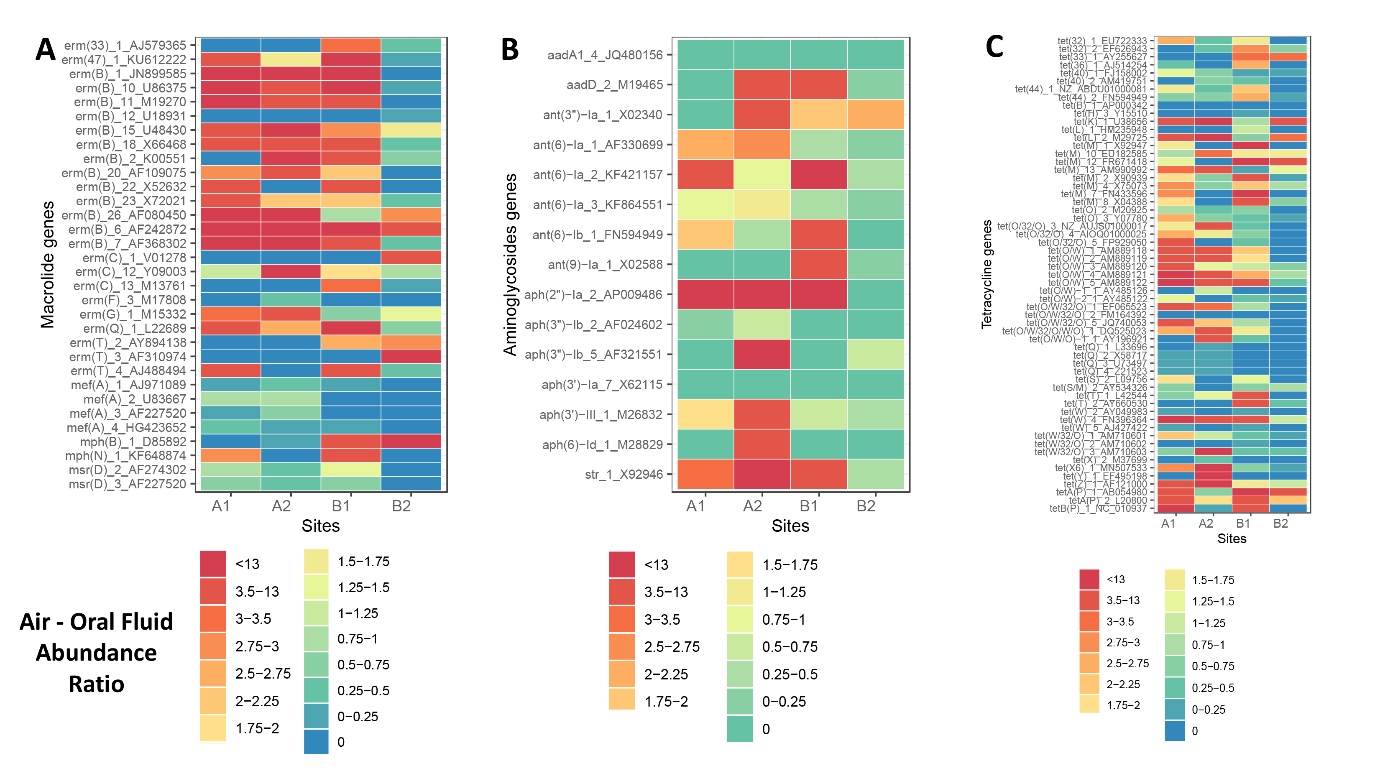


**Fig S7. Air to oral fluid ratios on antimicrobial resistance genes.** Warmer colours (orange to red) indicate a higher abundance air to oral fluid ratio (1>) which means the abundance of the given bacteria is greater in air compared to oral fluid samples. Cooler colours i.e. blue to green (1<) indicate a low abundance ratio, meaning the abundance of a given bacteria is higher in oral fluid samples compared to air samples. When the ratio is 0, the bacteria is present in only one sample type, while when the ratio is equal to 1, the bacteria are equally abundant in both samples. **6A.** indicates genes that encode resistance for macrolides. **6B.** shows genes that encode for aminoglycosides and **6C.** indicates tetracycline.
